# Molecular and cellular effects of gold nanoparticles treatment in experimental diabetic myopathy

**DOI:** 10.1016/j.heliyon.2022.e10358

**Published:** 2022-08-21

**Authors:** Aseel Al-Shwaheen, Alaa A.A. Aljabali, Ghada Alomari, Mazhar Al Zoubi, Walhan Alshaer, Bahaa Al-Trad, Murtaza M. Tambuwala

**Affiliations:** a Department of Biological Sciences, Faculty of Science, Yarmouk University, Irbid, 211-63, Jordan; b Department of Pharmaceutics and Pharmaceutical Technology, Faculty of Pharmacy, Yarmouk University, Irbid, Jordan; c Department of Basic Medical Sciences, Faculty of Medicine, Yarmouk University, Irbid, 211-63, Jordan; d Cell Therapy Center, University of Jordan, Amman, 11942, Jordan; e School of Pharmacy and Pharmaceutical Sciences, Ulster University, Coleraine, BT52 1SA, County Londonderry, Ireland

**Keywords:** Diabetes mellitus, Gold nanoparticles, Diabetic myopathy, Skeletal muscle, Nanomedicine

## Abstract

**Background:**

This study aims to address the effects of gold nanoparticles (AuNPs) on diabetic myopathy in streptozotocin (STZ)-induced diabetic rats.

**Materials and methods:**

Adult male rats were separated into three groups (n = 15): non-diabetic control (ND), diabetic (D), and diabetic treated with AuNPs (2.5 mg/kg, D + AuNPs) intraperitoneally for 4 weeks. A single injection of 50 mg/kg STZ was used to induce diabetes.

**Results:**

Treatment with AuNPs lowered blood glucose levels. Skeletal muscle mRNA expression of two muscle-specific E3 ubiquitin-ligases enzymes, F-box-only protein 32 (FBXO32) and muscle RING-finger protein-1 (MuRF1) were upregulated in the D group. Diabetic rats showed significant increases in the skeletal muscle expression levels of plasminogen activator inhibitor-1 (PAI-1), tumor necrosis factor-α (TNF-α), transforming growth factor-β1 (TGF-β1), and a decrease in glucose transporter 4 (GLUT4) expression. Superoxide dismutase (SOD) activity decreased and malondialdehyde (MDA) level increased in skeletal muscles of D group. Compared to the D group, expression levels of FBXO32, MuRF1, PAI-1 TNF-α, and TGF-β1 were decreased in the D + AuNPs group, and mRNA of GLUT4 increased. Furthermore, in D + AuNPs group, skeletal muscle MDA levels decreased while SOD activity increased.

**Conclusion:**

In experimental models, AuNPs can ameliorate muscle atrophy by reducing hyperglycemia, inflammation, and oxidative stress, and by suppressing the ubiquitin-proteasome proteolytic process.

## Graphical abstract

**Figure:**
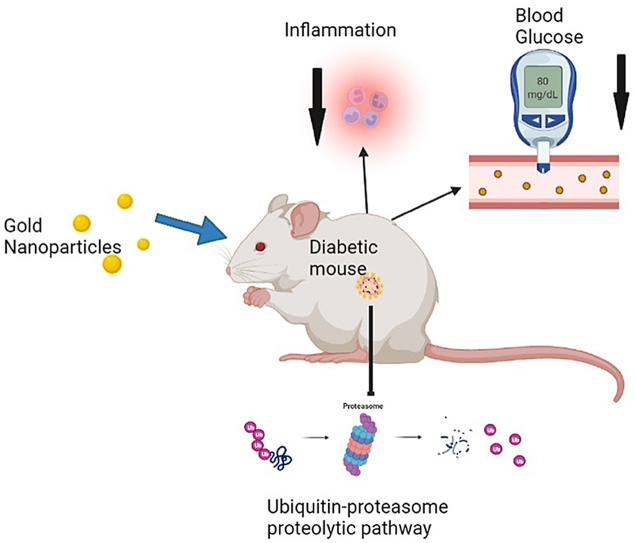

## 1 Introduction

Diabetes mellitus (DM) is a state of hyperglycemia associated with metabolic disease due to impaired insulin release and/or function [1]. Currently, DM is a global health problem associated with many complications that affect the healthy lives of patients [2]. Preventions and management of microvascular (retinopathy, nephropathy, and neuropathy) and macrovascular complications (diseases of the coronary arteries, peripheral arteries, and cerebrovasculature) associated with diabetes are major goals of care for DM [3]. However, DM is also associated with various diseases other than vascular complications. Diabetic skeletal muscle atrophy, defined by decreased skeletal, and muscular strength, and mass, is a common complication of diabetes called diabetic myopathy [1]. Diabetic myopathy contributes to the development of complications and other comorbidities of diabetes [4] and is considered a significant clinical challenge associated with a reduced quality of life [5].

Skeletal muscle is the largest body component in humans, accounting for more than 40% and 30% of the total body mass in men and women, respectively [6]. Skeletal muscle is involved in several physiological activities, including thermogenesis, metabolism, and the production of various peptides to communicate with other tissues and maintain an upright posture and produce movement [7]. As the largest organ, the Skeletal muscle is one of the most important tissues that maintain glucose homeostasis. The postprandial state is the main site of insulin-stimulated glucose uptake mediated by glucose transporter type 4 (GLUT4) translocation [8].

Several studies have investigated the link between DM and skeletal muscle mass and strength decline, i.e., muscle atrophy [1, 9]. Oxidative stress and low-grade chronic inflammation play an important role in the underlying mechanisms for poor muscle function in diabetes [1, 10]. Activation or inactivation of several intracellular signaling pathways during the state of oxidative stress and chronic inflammation will induce apoptosis and impaired muscle progenitor cell (muscle satellite cell) myogenic capacity and extracellular matrix (ECM) remodeling, which cause the primary pathology of the significant loss of muscle mass [1]. Activating the ubiquitin-proteasome proteolytic pathway of the ubiquitin-proteasome through increased expression of the E3 ubiquitin-ligase enzymes F-box-only protein 32 (FBXO32) and muscle RING-finger protein-1 (MuRF1) are responsible for the increase in muscle proteolysis. This contributed to the loss of muscle mass and myofibrillar proteins in various muscle-wasting conditions, including diabetes [11]. In addition, an increase in the skeletal muscle plasminogen activator inhibitor-1 (PAI-1) levels, an inhibitor of the fibrinolytic system, can impair ECM remodeling and eventually delay muscle regeneration capacity after muscle damage in type 1 DM [12]. Previous results demonstrated that oxidative stress and elevated levels of transforming growth factor-β (TGF-β) and tumor necrosis factor-α (TNF-α) had been implicated in increasing ubiquitin proteolytic activity through the expression of the E3 ubiquitin-ligases genes FBXO32 and MuRF1 and in decreasing fibrinolytic pathway activity in different skeletal muscle myopathies, including diabetic myopathy [[13], [14]].

Recently, the pharmaceutical industry has shown interest in nanotechnology-based drug development. Nanomedicine has been a vital effect in treating various fatal diseases. AuNPs are an example of nanomaterials that are convenient and suitable particles with unique optical, physical, and electrical properties. AuNPs have low toxicity, are biocompatible with the body, have a large surface-to-volume ratio, and can be functionalized [15, 16]. Therefore, and among other nanoparticles, AuNPs received great attention. Previous studies have shown that AuNPs exhibit anti-inflammatory [17], antioxidant [18], anti-angiogenic [19], antiproliferative [20, 21], and anti-diabetic [22] effects in different disease models. Chronic treatment of AuNPs in rats with a traumatic muscle injury reduced inflammation improved histological alterations, and increased antioxidant activity in skeletal muscle [17]. Moreover, AuNPs enhance myogenic differentiation and skeletal muscle regeneration capacity *in vitro* and *in vivo* [23]. Considering these promising results, we hypothesized that AuNPs would protect against the development of skeletal muscle atrophy in streptozotocin (STZ)-induced diabetic rats.

## 2 Material and methods

### 2.1 Synthesis and characterization of gold nanoparticles

AuNPs were prepared by the sodium citrate reduction method. Initially, 30 mL of ultra-pure H_2_O and 300 μl of a freshly aqueous solution of 1% (w/v) tetrachloroauric acid (HAuCl_4_) (Sigma–Aldrich) were added to a 250 mL Erlenmeyer flask (tightly cleaned with a copious amount of water). The solution was rapidly brought to a boil, stirring gently at 150 rpm at ambient temperature. Once the solution came to a boil (it took approximately 15 min), 900 μl of fresh aqueous 1% (*w/v*) sodium citrate trihydrate solution (Sigma-Aldrich) was added to the reaction mixture. The reaction was allowed to proceed for 10 min, during which time the formation of AuNPs was completed, and the color of the solution changed from yellow to ruby-red [21].

UV-vis spectrophotometry was used as an indicator of reaction completeness, using a Multiskan GO spectrophotometer (Thermo Scientific, USA) with a resolution of 2 nm and a wavelength range (240–800 nm). The measurements were taken at 4-hour intervals at room temperature in a 1 cm long quartz cuvette followed until the reaction was complete.

The Zetasizer Nano ZS90 (Malvern Panalytical, UK) was used to measure the surface charge, and hydrodynamic diameter of the formed nanoparticles, in which 100 μl of the solution was suspended in 900 μl distilled water. Then three independent measurements were made under the following conditions: 25 °C, 1.33 dispersant refractive index, and 0,8872 cP viscosity. Each measurement was set for 10 runs of 30 s per run. Characterization of the shape of the nanoparticles formed through TEM was achieved using an FEI Titan Transmission Electron Microscope (FEI company Hillsboro, OR, USA), in which the sample was first dispersed in water then deposited on a carbon grid, and then allowed to dry before imaging.

### 2.2 Induction of type 1 diabetes in rats and treatment

All experimental procedures were approved by the Institutional Animal Care and Use Committee at Yarmouk University (IACUC/2021/3). This study used adult male Sprague-Dawley rats, 55–60 days old and weighing about 250–300 g. All animals were housed at the Animal House at Yarmouk University/Jordan in a controlled environment with a room temperature of 22 °C ± 2 °C on an illumination schedule of 12 h of light and 12 h of darkness. Standard pellet food and water were provided *ad libitum*. A single intraperitoneal injection of freshly produced streptozotocin (STZ; 50 mg/kg; dissolved in 0.1 M acetate solution; pH 4.5) was used to develop diabetes in rats. Fasting blood glucose levels were evaluated three days after injection using a glucometer (GlucoLab, Infopia, Korea), and rats with blood glucose levels greater than 150 mg/dL were considered diabetic. The rats were then divided into three groups (each with 15 rats): (1) non-diabetic control rats (ND) were given tap water only for four weeks; (2) diabetic rats (D) were given tap water only for four weeks; and (3) diabetic rats were given 2.5 mg/kg gold nanoparticles intraperitoneally for four weeks [24]. At the end of the experiment, the animals were weighed and anesthetized with ether, and blood was collected (by cardiac puncture). The lower limb muscles were removed and divided into two symmetrical parts. The first part was snap-frozen in liquid nitrogen for molecular and biochemical investigation, while the second part was fixed in 10% buffered formaldehyde for morphological analysis.

## 3 Blood glucose measurement

According to the manufacturer's protocol, blood glucose levels were measured in serum samples using the colorimetric GOD-PAP method (Fortress Diagnostics, UK).

### 3.1 Histopathological examination

After the fixation in 10% buffered formaldehyde, muscle tissues were dehydrated in ascending series of alcohol, cleared in xylene, embedded in paraffin, sectioned at 8 μm, and stained with hematoxylin and eosin (H&E). Sections were examined and visualized under a B-150 DB optical microscope (Optika, Italy). Cross-sectional myocyte areas of the lower limb muscle were measured for at least 150 fibers per animal. Image J version 1.48 was used for these analyzes.

#### 3.1.1 Real-Time PCR (RT-PCR)

Total RNA was extracted using the TRIzol™ Reagent method. A cDNA template was prepared using a cDNA reverse transcription kit (Takara, Japan). Quantitative real time-RT-PCR was conducted using thermocycler CFX96 Real-Time PCR systems (CFX96; Bio-Rad, USA) using BlasTaq™ 2X qPCR MasterMix (Applied Biological Materials, Canada). Cycling parameters were: 95 °C for 3 min and 40 cycles of 95 °C for 5 s and 60 °C for 30 s. Relative mRNA concentrations were normalized for the GAPDH housekeeping gene. The primer sequences used in the reaction are shown in Table 1. The fold changes in mRNA expression were determined using the 2^−ΔΔCT^ method [25].

**Table 1:** The sequence of the primers used for RT-PCR.

Gene	Primer sequence (5’ -3′)	Reference
GAPDH	F: ATGGTGAAGGTCGGTGTG	[26]
	R: GAACTTGCCGTGGGTAGA	
TGF β1	F: GTGGAGCAACACGTAGAAC	[24]
	R: TCCTTGGTTCAGCCACT	
TNF α	F: TTCGGAACTCACTGGATCCC	[24]
	R: GGAACAGTCTGGGAAGCTCT	
GLUT 4	F: CCCCCGATACCTCTACAT	[27]
	R: GCATCAGACACATCAGCCCAG	
PAI 1	F: GACAATGGAAGAGCAACATG	[28]
	R: ACCTCGATCTTGACCTTTTG	
FBXO32	F: CAACATGTGGGTGTATCGAATGG	[29]
	R: TGATGTTCAGTTGTAAGCACACAGG	
MuRF1	F: GTGAAGTTGCCCCCTTACAA	[30]
	R: TGGAGATGCAATTGCTCAGT	

**Abbreviations:** GAPDH: Glyceraldehyde 3-phosphate dehydrogenase; TGF-β1: Transforming growth factor beta; TNF-α: Tumor necrosis factor-α; GLUT4: Glucose transporter type 4; PAI-1: Plasminogen activator-inhibitor-1; FBXO32: F-box-only protein 32; MuRF1: Muscle RING finger 1.

### 3.2 Determination of the oxidative stress markers

Superoxide Dismutase (SOD) activity calorimetrically using a superoxide dismutase assay kit (Abbkine, China). The malondialdehyde level of skeletal muscle tissue (MDA), a lipid peroxidation marker, was assessed by a method described by Buege and Aust [31].

### 3.3 Statistical analysis

The data are presented with a standard error mean (SEM). The one-way analysis of variance (ANOVA) test was used to perform the data analysis using SPSS version 26 (SPSS Inc., Chicago, IL), and statistical significance was considered when the *P*-value < 0.05.

## 4 Results

### 4.1 AuNPs characterization

**Figure 1:**
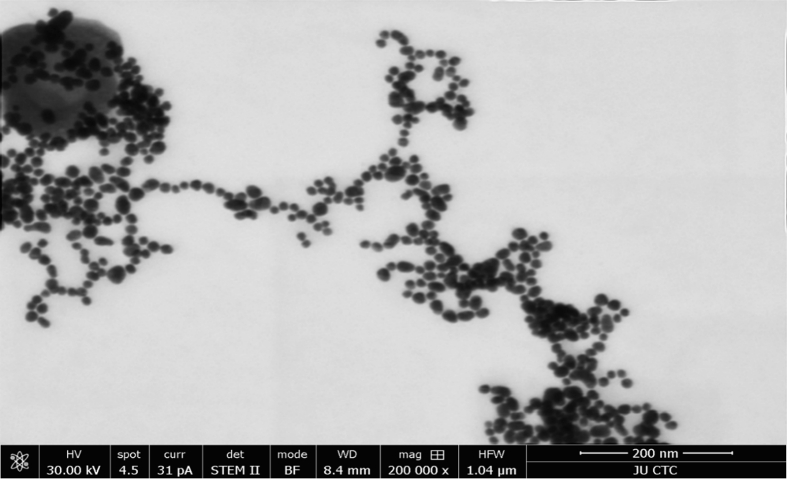
A representative unstained TEM image of the formed AuNPs.

The generated AuNPs mainly were spherical, ruby-red color, about 15–50 nm in size, with good uniformity in the size distribution of the AuNPs, as confirmed by the TEM image of AuNPs in Figure 1. The AuNPs were analyzed by UV spectrophotometer absorbance spectra, as shown in Figure 2.1, where the spectra of AuNPs synthesis showed an increase in the Surface Plasmon Resonance (SPR) excitation peak centered at 524 nm confirming the formation of AuNPs has been previously reported [16].

**Figure 2:**
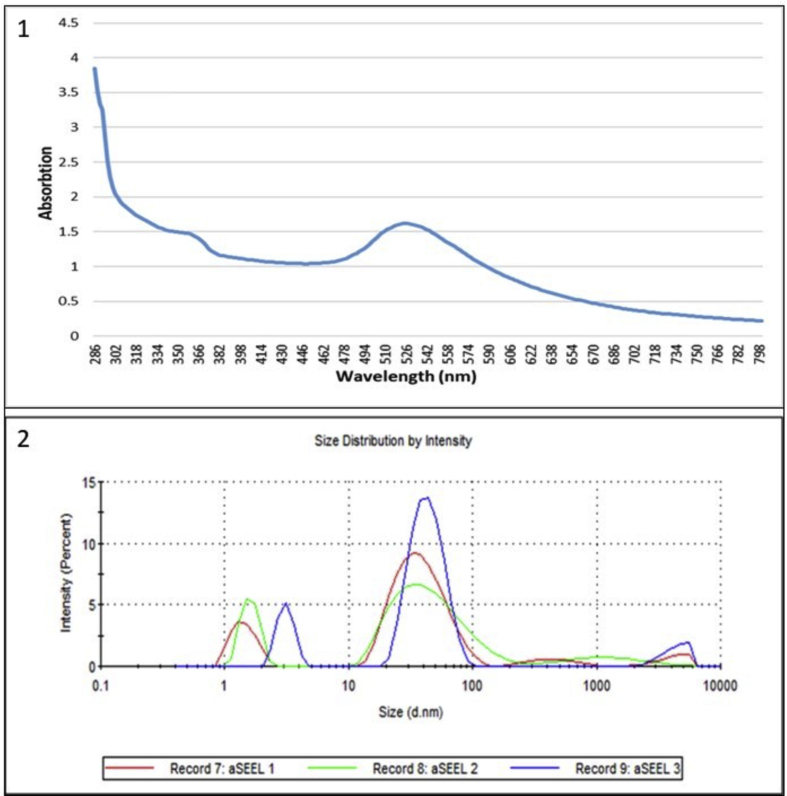
(1) UV-visible absorption spectra of AuNPs; (2) Size distributions of AuNPs as determined by DLS.

Further characterization was carried out to determine the particle size distributions by determining the hydrodynamic diameter of the nanoparticles in a liquid suspension by dynamic light scattering (DLS). Figure 2.2 shows the DLS analysis for the synthesis of AuNPs, with the average size (21.7 nm) falling within the expected range of particle sizes between 15 and 50 nm, which is very similar to the size observed in TEM (21 nm), with the polydispersity index (PDI) of 0.623, in addition, the Zeta potential (ζ) measurements showed that the surface charge of the nanoparticle was −26.2 mV.

### 4.2 Rats weight and blood glucose levels

A significant decrease in body weight was observed in the D group at the end of the experiment compared to the beginning of the experiment of the same group (*P* < 0.05; Figure 3.1). No significant changes in the body weight were observed in the ND and D + AuNPs groups (Figure 3.1). After 4 weeks of STZ injection, D rats showed significant increases in blood glucose compared to the ND group (*P* < 0.05; Figure 3.2). However, treating diabetic rats with AuNPs significantly lowered the blood glucose level compared to the D group (*P* < 0.01; Figure 3.2).

**Figure 3:**
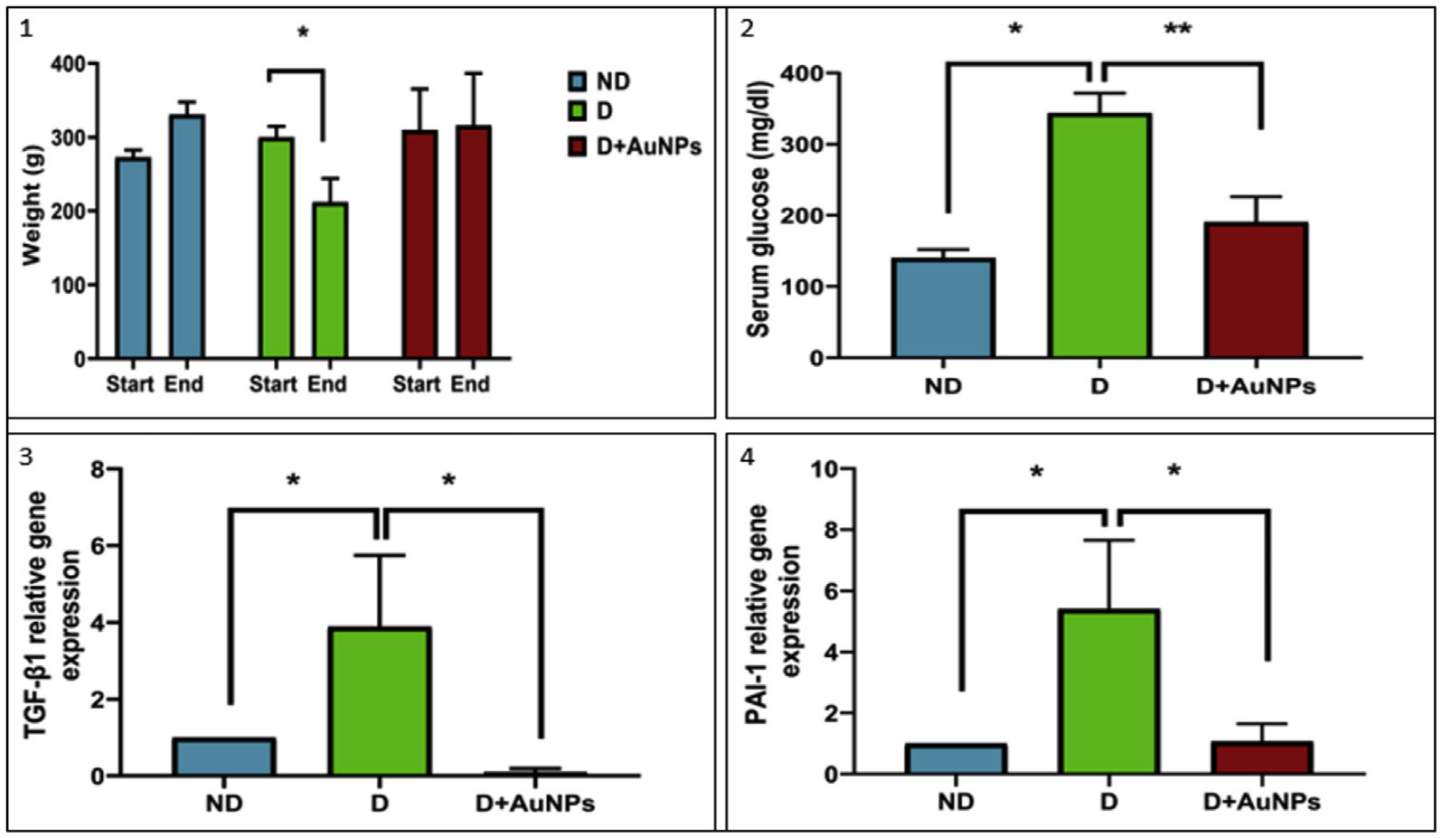
(1) Weight of Rats (g) at the beginning and end of the experiment; (2) Blood glucose level (mg/dl) expressed as mean ± SE; (3) The relative gene expression of TGF-β1 among experimental groups; (4) The relative gene expression of PAI-1 among experimental groups. (ND: Non-Diabetic group, D: Diabetic group, D + AuNPs: Diabetic treated with AuNPs, ∗*P* < 0.05, ∗∗*P* < 0.01).

### 4.3 Histopathological changes

**Figure 4:**
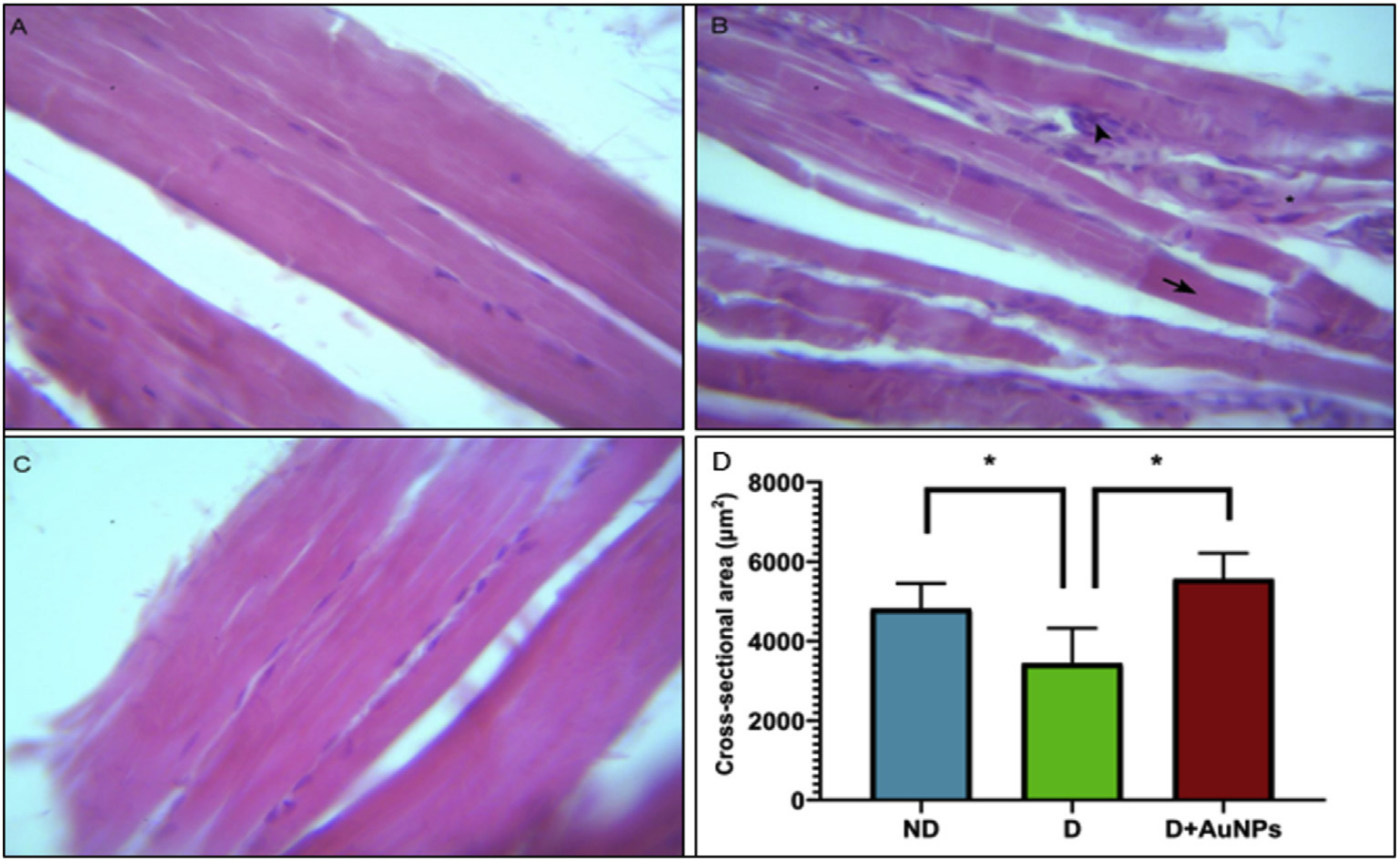
Histopathological changes in the lower limb muscles in all groups of rats. (A) ND rat. (B) D rat. (C) D + AuNPs rat. (D) Data of myocyte cross-sectional area of the lower limb muscles from groups (n = 8 each); Showing in figure B, an area of endomysial inflammation (arrowhead), necrosis of the myofibers (arrow), and degenerated fiber de (astrisk). (Magnification ×400). Scale bars represent 10 μm. Data are means ± SEM. ∗*P* < 0.05).

Histological examination of H&E-stained sections of the lower limb muscles of the ND group (Figure 4A) showed adequately preserved myofibers with clear striation and peripheral myonuclei. Areas of inflammation infiltrating muscles fibers, mainly in the endomysial layer of skeletal muscle, necrotic processes in the fibers, and loss of the cross-striations and phagocytosis were seen in the STZ-diabetic rats (Figure 4B). However, treatment with the AuNPs reduced the above histological abnormalities, myofibers arranged in neat rows, striations, and eccentrically located nuclei were clear, and the soak of inflammatory cells was less, as shown in (Figure 4C).

The myocyte cross-sectional area of the lower limb muscle was significantly smaller in the D group than in the ND group (*P* < 0.05; Figure 4D) and was significantly improved in the D + AuNPs group compared to the D group (*P* < 0.05; Figure 4D).

### 4.4 Effect of AuNPs on the selected genes expression levels

Furthermore, compared to the ND group, there were significant increases (*P* < 0.05) in the expression of TGF- 1 skeletal muscle mRNA expression of TGF-β1 (Figure 3.3), PAI-1 (Figure 3.4), TNF-α (Figure 5.1), FBXO32 (Figure 5.2), MuRF1 (Figure 5.3), and a decrease in the expression of GLUT4 (Figure 5.4) in the D group. Compared with the D group, the mRNA expression levels of FBXO32, MuRF1, PAI-1, TNF-α, and TGF-β1, were significantly decreased *P* < 0.05) in the D + AuNPs group, and the mRNA of GLUT4 was significantly increased.

**Figure 5:**
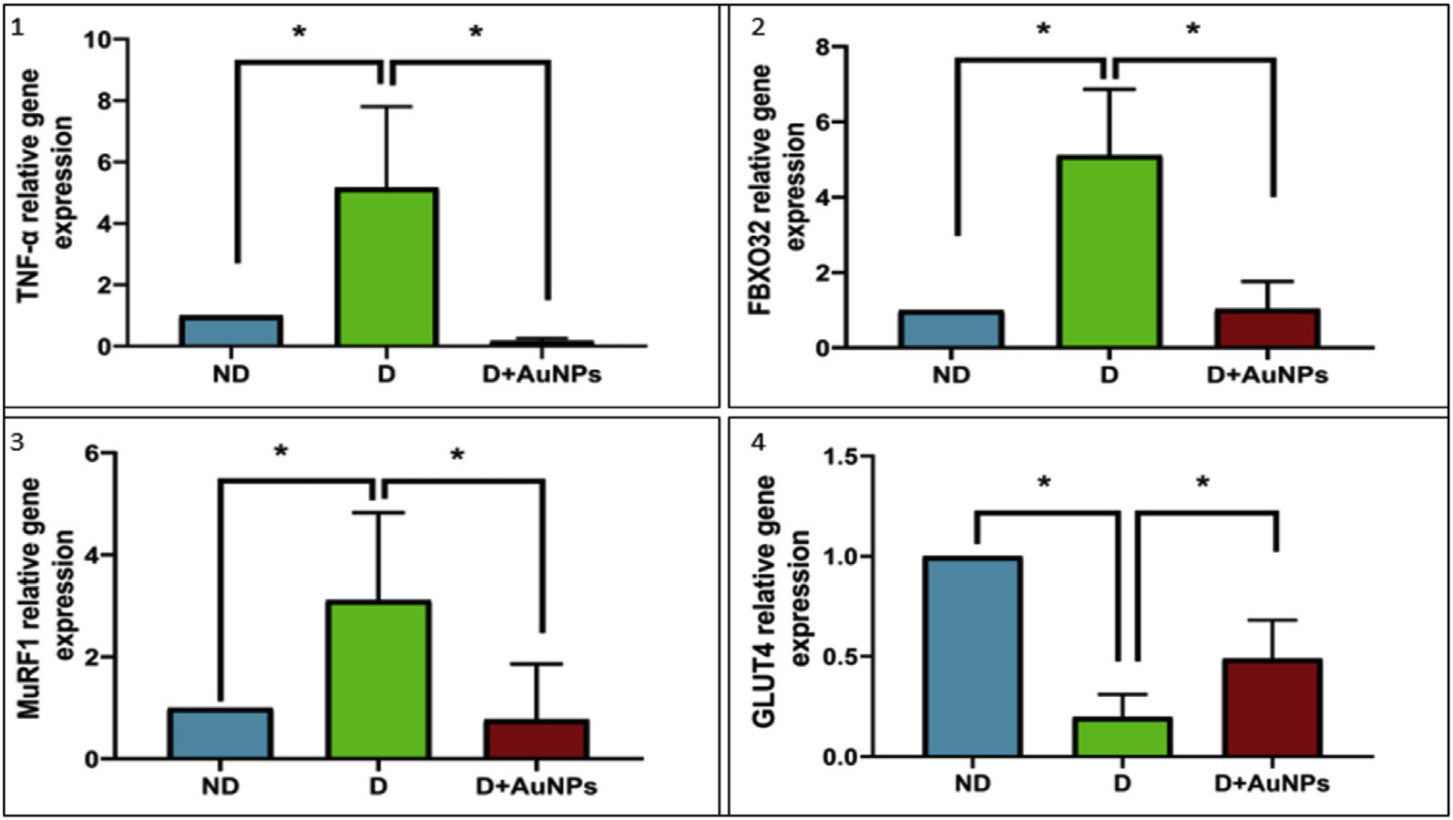
The relative gene expression of: (1) TNF-α; (2) FBXO32; (3) MuRF1; (4) GLUT4 among experimental groups. (ND: Non-Diabetic group, D: Diabetic group, D + AuNPs: Diabetic treated with AuNPs, ∗*P* < 0.05).

### 4.5 Effect of AuNPs on the oxidative stress markers

The diabetic group showed a significant increase in muscle tissue MDA levels and decreased SOD activity compared to the ND group (*P* < 0.05; Figures 6.1,2). In particular, the D + AuNPs group showed a significant decrease in the muscle tissue MDA levels (*P* < 0.01; Figure 6.1) and a tendency to increase in the muscle tissue SOD activity (*P* < 0.1; Figure 6.2) compared to the D group.

**Figure 6:**
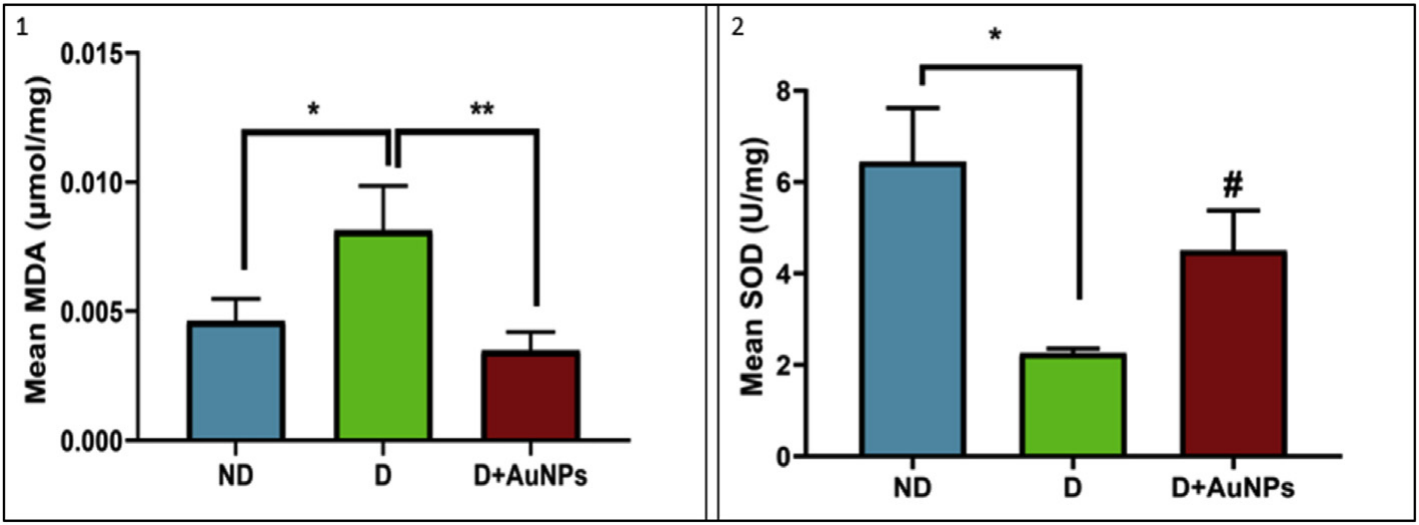
Effects of AuNPs on: (1) malondialdehyde (MDA) levels; (2) superoxide dismutase (SOD) activity level in skeletal tissue. (ND: Non-Diabetic group, D: Diabetic group, D + AuNPs: Diabetic treated with AuNPs, ∗*P* < 0.05, ∗∗*P* < 0.01, #*P* < 0.1).

## 5 Discussion

A reduction in skeletal muscle physical ability, strength, and mass is often observed in DM [1]. Therefore, it is important to look for effective therapy to address skeletal muscle atrophy in diabetes. Nanomaterials have been used in many biomedical applications for their unique properties [32], among which AuNPs have received great attention due to their unique properties and as a significant antidiabetic agent [18, 24]. Researchers have shown the benefits of AuNPs on different types of myopathy [17, 33]. However, the literature has not described the possible protective effect of AuNPs against the development of skeletal muscle atrophy in STZ-induced diabetes.

Type 1 DM is one of the most common clinical conditions in which muscle growth capacity and performance can be significantly altered [34]. Rats are sensitive to the cytotoxic effects of STZ, an antibiotic that produces β-cell destruction of -cells in the pancreas. Therefore, STZ is widely used experimentally to induce animal models for type 1 DM [35]. A recent study demonstrated that STZ-induced type 1 DM is suitable for studying the molecular mechanism of diabetic myopathy in animal models [36]. Diabetic rats used in this study exhibit hyperglycemia, elevated levels of pro-inflammatory cytokine skeletal muscle mRNA expression (TNF-), MDA levels of proinflammatory cytokine (TNF-α), and atrophy-related genes (FBXO32 and MuRF1) MDA levels (a marker of oxidative stress), as well as muscle fiber atrophy as indicated by histological assessment. Therefore, we successfully established an experimental animal model of type 1 DM possessing diabetic myopathy.

The AuNPs used in this study were mainly spherical, with sizes ranging from 15–50 nm, considered less toxic and safe for medical applications [37]. The surface charge was −26.2 mV, indicating good stability of the formed nanoparticles and low aggregation potential [38].

Hyperglycemia, a key feature of diabetes, plays a pivotal role in developing several diabetes complications [39], including diabetic myopathy [36]. General mechanisms of hyperglycemia-mediated pathophysiological complications and organ dysfunction include enhancing oxidative stress, upsurging polyol pathway, activating protein kinase C (PKC), and enhancing hexosamine biosynthetic pathway (HBP), promoting the formation of advanced glycation end-products (AGEs), and finally altering gene expressions [39]. Thus, glycemic management of diabetes remains the main target of the therapy. Consistent with previous study [40] and our past results [24, 41, 42], the present study confirmed that AuNPs possess a hypoglycemic effect in diabetic animal models. Several mechanisms have been suggested for the hypoglycemic effect exerted by AuNPs. AuNPs potentiate the effect of insulin in plasma by increasing the pancreatic secretion of insulin from Langerhans [43]. Other studies have shown that it has an inhibitory effect on certain digestive enzymes, including α-amylase and α-glucosidase [44], resulting in a decrease in glucose release. Recently, we showed that AuNPs synthesized using the leaf extract of *D. viscosa* can alleviate hyperglycemia in high-fat diet/STZ-induced diabetes in rats, which could be by reducing hepatic gluconeogenesis by inhibiting hepatic PEPCK gene expression and activity [41].

Skeletal muscle is the predominant site of insulin-mediated glucose uptake in the postprandial state via the GLUT4 and the major glucose transporter protein expressed in skeletal muscle [45, 46]. Insulin resistance and/or diabetes are associated with reduced skeletal muscle GLUT4 expression, and translocation leads to decreased skeletal muscle glucose uptake and impaired glycemic homeostasis [45]. Hence, increasing muscle-specific GLUT4 expression by enhancing insulin sensitivity can improve glucose homeostasis [45]. The AuNPs improved insulin-dependent glucose uptake activity in L6 rat skeletal muscle cell lines [46]. In addition, AuNPs significantly upregulated hepatic and retroperitoneal fat mRNA expression levels, contributing to the improved insulin response and glucose uptake in mice fed a high-fat diet [40]. In GLUT4 present study, the gene expression was reduced in the skeletal muscle of the D group. However, because AuNPs treatment significantly reversed downregulation of GLUT4 in the diabetic muscle, we postulated that the hypoglycemic action of AuNPs may be related, at least in part, to improved insulin sensitivity and increased glucose uptake in diabetic muscles. This hypothesis needs to be tested by further studies.

Muscle atrophy occurs due to increases in muscle fiber proteolysis when protein breakdown exceeds protein synthesis. The activation of the ATP-dependent, ubiquitin-proteasome pathway is mainly responsible for increasing muscle proteolysis in various muscle-wasting conditions, including diabetes [11, 14]. The process of protein ubiquitination is controlled by the hierarchical action of three general families of ubiquitin enzymes, Ub-activating enzymes (E1), Ub-conjugating enzymes (E2), and Ub-protein ligases (E3) [11]. An increase in the two E3 ubiquitin ligases, MuRF1, and FBXO32 mRNA expression, has been elevated in a wide range of atrophy-inducing conditions and prior to the onset of muscle loss which has become recognized as key markers of muscle atrophy [11, 47]. In diabetic conditions, oxidative muscle damage and the proinflammatory microenvironment increase the expression levels of the two atrophy-associated genes, MuRF1, and FBXO32, which shift protein balance from net synthesis to net degradation and ultimately loss of muscle mass [11, 48]. Previous studies have revealed that mice deficient in MuRF1 and/or FBXO32 will be more resistant to muscle atrophy induced by denervation or glucocorticoid treatment [11, 49]. In the current study, the gene expressions of MuRF1 and FBXO32 in skeletal muscle were increased in the D group. However, the treatment of AuNPs significantly reversed the upregulation of MuRF1 and FBXO32 in diabetic muscle, and thus we concluded that AuNPs could partially inhibit the ubiquitin-mediated proteolysis pathway by reducing the expression of FBXO32 and MuRF1.

Several cytokines induce muscle atrophy, most notably TNF-α, a significant mediator of the inflammatory process. It has also been reported that TNF-α is associated with the development of diabetes and can also alter insulin-mediated glucose uptake in muscle cells *in vitro* [1]. TNF-α is also responsible for increased expression of ubiquitin and accumulation of circulating proteins such as MuRF1 [49] and FBXO32 [11]. TNF-α is considered a producer and activator of NF-κB (nuclear factor kappa-light-chain-enhancer for activated B cells), thus initiating a positive feedback loop that enhances the expression of NF-κB and exacerbates muscle degradation [50]. *In vivo*, TNF-α induces myotube atrophy with overexpression of FBXO32 in mouse skeletal muscle C2C12 cells [51]. TNF-α-activated NF-κB has also been found to regulate MuRF1 [50]. Previous studies demonstrated that AuNPs are anti-inflammatory in muscle injury models by reducing the levels of cytokines IL-1β and TNF-α in injured muscle [33]. Treatment with AuNPs also induced a significant decrease in the mRNA expression levels of TNF-α in the diabetic nephropathy model, which suppressed the inflammation [24]. Furthermore, AuNP treatment reduced myocardial mRNA and protein levels of TNF-α in diabetic cardiomyopathy (DCM), resulting in a reduced intramyocardial inflammatory response and cardiac collagen content [42]. In this study, TNF-α gene expression was increased in diabetic skeletal muscle. However, the treatment of AuNPs reduced skeletal muscle TNF-α gene expression levels in diabetic rats.

In diabetic patients, ECM accumulation and fibrosis occur through fibroblasts that produce TGF-β1, which promotes the synthesis and accumulation of ECM components and contributes to the development of fibrosis, followed by atrophy of muscle fibers. TGF-β1 also improves collagen synthesis and inhibits the expression of ECM-degrading proteases, such as matrix metalloproteinases (MMPs) [52]. TGF-β1 Inhibition through the use of neutralizing antibodies against TGF-β1 and TGFβ-receptor I (TβRI) has been shown to significantly improve muscle regeneration accompanied by reduced progression of fibrosis [53]. TGF-β1 was also inhibited by insulin-like growth factor-1 (IGF-1) treatment, which prevents skeletal muscle fibrosis by inhibiting TGF-β1-induced SMAD phosphorylation, which leads to decreased accumulation of ECM components [54]. Recently, treatment with AuNPs has reduced the renal and cardiac expression of TGF-β1 in experimentally induced diabetic nephropathy [24], and cardiomyopathy [42]. In the present study, AuNPs treatment significantly decreased TGF-β1 levels in diabetic skeletal muscle; therefore, we speculate that one of the molecular mechanisms that underlie the protective effect of AuNPs against diabetic skeletal muscle fibrosis and damage is likely mediated by the inhibition of TGF-β1 expression and signaling pathway.

Plasmin directly or indirectly reduces the accumulation of ECM by activating latent MMPs. Its expression is primarily regulated by the serine protease inhibitor, PAI-1, which inhibits both plasminogen activator, tissue-type plasminogen activator (tPA), and urokinase-type plasminogen activator (uPA) and thus reduces plasmin generation [12]. Plasminogen activator inhibitor-1 (PAI-1) is the primary regulator of the plasminogen system that exacerbates various disease states through ECM accumulation (i.e., fibrosis), as well as its role in altering cell fate/behavior [55]. In type 1 DM, the remodeling of the ECM is impaired, which is a result of elevated PAI-1 [55], which in turn leads to decreased levels of active uPA and MMP9, thus reducing ECM turnover (as noted by elevated intramuscular collagen) during the first 10 days after injury [12]. PAI-1 plays a unique role in response to skeletal muscle injury and myopathy, highlighting the importance of the plasminogen system and the remodeling of the ECM of skeletal muscle [12, 55]. Restoration of the fibrinolytic system in mice with type 1 DM by pharmacological inhibition of PAI-1 restored active expression of MMP9, returned collagen levels to normative values, and ultimately allowed nascent muscle fiber growth to occur [12]. In a recent study, PAI-1 secreted levels were decreased significantly through AuNPs-loaded macrophages [56]. In the current study, AuNP treatment showed a significant decrease in PAI-1 mRNA expression of PAI-1 in skeletal muscle and, as a result, restored active expression of MMP9, thus helping to degradation and remodeling and growth of emerging muscle fibers.

Hyperglycemia stimulates mitochondrial respiration, which causes the liberation of reactive oxygen species (ROS) in large quantities into the cytoplasm, and thus leads to oxidative stress. Oxidative stress induced by ROS or failure of the antioxidant defense mechanism is an essential factor in the activation of different signaling pathways involved in the development of diabetes and its complications, including myopathy [57]. Therefore, the neutralization of ROS caused by hyperglycemia is sufficiently adequate for preventing experimentally induced diabetes and its complications. The antioxidant effect of AuNPs is well documented in various studies [[24], [33]]. In the current study, the antioxidant effects of AuNPs were evaluated by measuring the lipid peroxidation product MDA. MDA is a byproduct of lipid oxidation by free radicals and ROS, and this aldehyde readily interacts with protein or DNA, forming adducts, and is highly genotoxic. Furthermore, SOD, an antioxidant enzyme that converts superoxide radicals to H_2_O_2_, plays an essential role in the defensive process that protects cells from ROS [58]. The results showed that AuNPs significantly improved the antioxidant defense mechanisms in diabetic skeletal muscle, as indicated by the significant decrease in the MDA level and the tendency to increase in the levels of SOD in the D + AuNPs group compared to the D group.

## 6 Conclusions

These results prove that AuNPs can ameliorate muscle atrophy in experimental models of diabetes by reducing hyperglycemia, inflammation, oxidative stress, and the proteolytic pathway. The present findings may help design the clinical application of AuNPs for protection against the development of skeletal muscle atrophy caused by diabetes.

## Declarations

### Author contribution statement

Aseel Al-Shwaheen: Performed the experiments; Analyzed and interpreted the data and; Wrote the paper.

Alaa A. A. Aljabali; Mazhar Al Zoubi: Contributed reagents, materials, analysis tools or data; Wrote the paper.

Ghada Alomari: Performed the experiments; Analyzed and interpreted the data.

Walhan Alshaer: Analyzed and interpreted the data; Wrote the paper.

Bahaa Al-Trad: Conceived and designed the experiments; Analyzed and interpreted the data; Contributed reagents, materials, analysis tools or data; Wrote the paper.

Murtaza M. Tambuwala: Conceived and designed the experiments; Analyzed and interpreted the data Wrote the paper.

### Funding statement

This work was supported by the Deans of Scientific Research and Graduate studies at Yarmouk University Grant number 46/2021.

### Data availability statement

Data will be made available on request.

### Declaration of interest statement

The authors declare no conflict of interest.

### Additional information

No additional information is available for this paper.
